# Static and Dynamic Magnetic Properties of Fe_3_O_4_ Nanotubes

**DOI:** 10.3390/nano13071265

**Published:** 2023-04-03

**Authors:** Francisco Olea de la Hoz, Eduardo Saavedra, Alejandro Pereira, Juan Escrig

**Affiliations:** 1 Department of Physics, University of Santiago de Chile (USACH), Santiago 9170124, Chile; 2 Department of Sciences, Faculty of Liberal Arts, Adolfo Ibañez University (UAI), Santiago 7941169, Chile; 3 Center for the Development of Nanoscience and Nanotechnology (CEDENNA), Santiago 9170124, Chile

**Keywords:** magnetic properties, Fe_3_O_4_, magnetic nanotubes, hysteresis curves, FMR

## Abstract

In this paper, our objective was to investigate the static and dynamic magnetic properties of Fe_3_O_4_ nanotubes that are 1000 nm long, by varying the external radius and the thickness of the tube wall. We performed a detailed numerical analysis by simulating hysteresis curves with an external magnetic field applied parallel to the axis of the tubes (along the *z*-axis). Our findings indicate that nanotubes with an external radius of 30 nm exhibit non-monotonic behavior in their coercivity due to a change in the magnetization reversal mechanism, which was not observed in nanotubes with external radii of 80 nm. Additionally, we explored the dynamic susceptibility of these nanotubes and found that the position and number of resonance peaks can be controlled by manipulating the nanotube geometry. Overall, our study provides valuable insights into the behavior of Fe_3_O_4_ nanotubes, which can aid in the design and improvement in pseudo-one-dimensional technological devices.

## 1. Introduction

When materials are reduced to the nanoscale, new properties emerge that differ from those exhibited by the same larger materials, as the characteristic dimensions of many physical phenomena are in the order of nanometers. These novel properties have enabled magnetic nanostructures to be utilized in various applications, including magnetic sensors, information storage systems, and spintronic-based devices, as well as medical applications such as hyperthermia or drug delivery. Going a step further, Niknam et al. [1] have recently proposed the use of nanomagnets for the quantum control of spin qubits, essential components of universal quantum computers. Proper control of the shape and size of these magnetic nanostructures can facilitate the design of innovative devices [2].

One of the most extensively studied magnetic nanostructures is magnetic nanotubes (MNTs) due to their high aspect ratio, similar to that of nanowires, but with two functionalizable surfaces [3]. Moreover, Yan et al. [4] have demonstrated that domain walls in a tubular geometry are more durable than those in flat strips, which can even suppress the Walker breakdown in these nanostructures. Bao et al. [5] proposed using ferromagnetic nanotubes as alternative skyrmion guides, which are a promising candidate for information carriers, while Yang et al. [6] investigated the stability of skyrmion formation in magnetic nanotubes. Additionally, Rojas-Nunez et al. [7] has shown that magnetic nanotubes provide a lightweight alternative for designing mechanical nanodevices with minimal loss of mechanical performance.

The field of magnetic nanotube synthesis has yielded a wide variety of materials that have been successfully utilized in the creation of these structures. Among the most commonly synthesized magnetic nanotubes are those made from materials such as iron (Fe) [8,9], Fe(OH)_3_ [10], maghemite (γ-Fe_2_O_3_) [10,11,12], magnetite (Fe_3_O_4_) [10,12,13,14,15,16,17,18], ZnFe_2_O_4_ [19], CuFe_2_O_4_ [20], nickel (Ni) [8,21,22,23], NiFe_2_O_4_ [24], Ni_64_Fe_36_ [8], Ni_80_Fe_20_ (permalloy) [25], Co [8,23,26], Co_3_O_4_ [27], Co_90_Pt_10_ [8], Co_75_Cr_13_Pt_12_ [8], and many others. In addition to these common materials, nanotubes with more complex geometries have also been synthesized, taking into consideration factors such as diameter modulations [28], multisegmented structures [29], and core–shell [30,31,32,33] systems. The synthesis of these advanced structures is a testament to the ingenuity and skill of researchers in this field, and it suggests that the possibilities for the creation of new and innovative magnetic nanotubes are practically limitless.

On the other hand, the static and dynamic magnetic properties of magnetic nanotubes, such as the magnetic equilibrium states [34,35,36,37,38], magnetization reversal mechanisms [16,17,21,23,37,38,39,40,41,42,43] and magnetostatic interaction [44,45,46,47], have also been investigated theoretically. González et al. [48] went further and calculated the spin wave spectra associated with the vortex domain wall confined within a nanotube, which encouraged the synthesis of Fe_3_O_4_ nanotubes with this type of domain wall [49]. Salazar-Cardona et al. [50] proposed a device based on coplanar waveguides that would selectively measure the exchange or dipole-induced spin wave nonreciprocities. Recently, some of us analyzed the dynamic susceptibility of curved permalloy nanotubes [51] and permalloy wire-tube nanostructures [52]. However, to the best of our knowledge, there are no studies that systematically investigate the static and dynamic properties of Fe_3_O_4_ nanotubes, despite the high aspect ratio being conducive to electron conduction and further increasing the conduction loss capability [53].

In this study, we investigate the static and dynamic magnetic response of long Fe_3_O_4_ nanotubes using micromagnetic simulations. Our objective is to examine how the magnetic properties vary with changes in the external radius and thickness of the tube wall. We focus particularly on the different magnetization reversal mechanisms and potential resonance modes that may appear.

## 2. Micromagnetic Simulations

**Figure 1:**
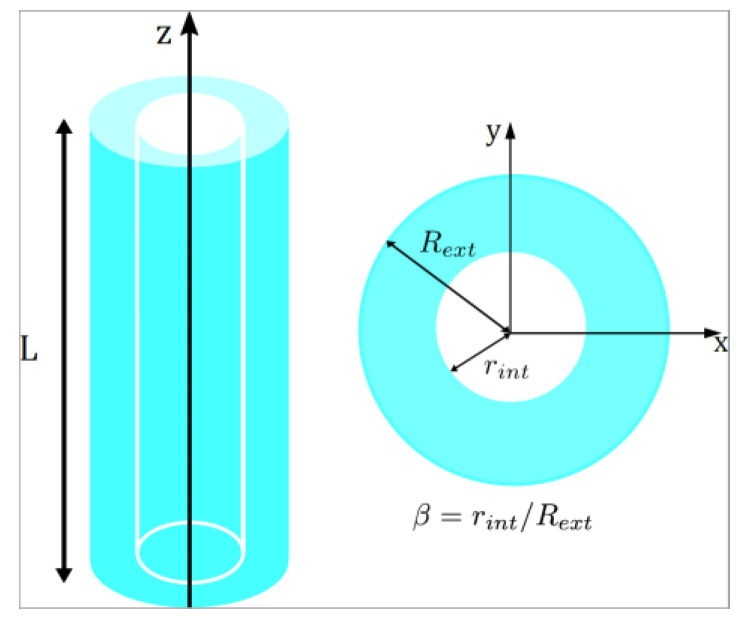
Schematic representation of an isolated magnetic nanotube. Representative geometric parameters are indicated.

The static and dynamic magnetic properties of a Fe_3_O_4_ nanotube (Figure 1) were studied using micromagnetic simulations performed using the mumax^3^ software [54], which solves the Landau–Lifshitz–Gilbert equation (LLG) [55]. The micromagnetic simulations were performed on a computer specifically configured to work with an RTX 3090 video card, which is ideal for running Mumax^3^ software in CUDA language. This RTX 3090 card has 10,752 CUDA cores, making it one of the best options for GPU-based micromagnetic studies.

**Figure A1:**
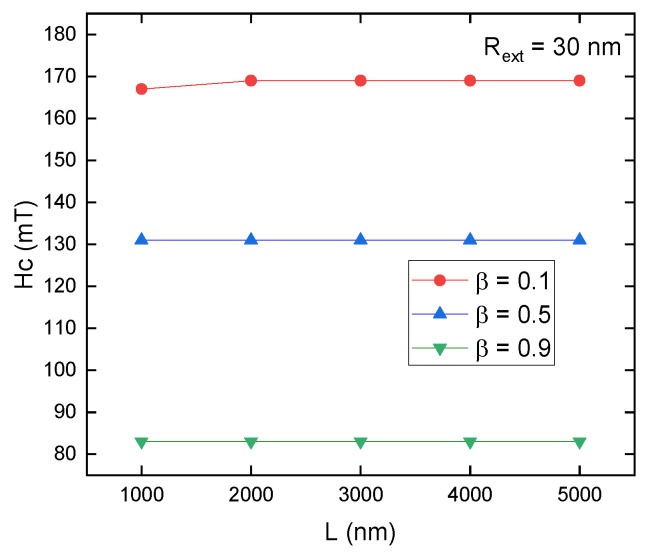
Coercivity for long Fe_3_O_4_ nanotubes with an external radius of 30 nm and three different hole sizes, *β* = 0.1, 0.5, and 0.9, with lengths ranging between 1000 and 5000 nm, when the external magnetic field is applied along the *z*-axis.

For spherical nanoparticles, there is a critical diameter that allows discriminating between a superparamagnetic phase and a single-domain phase, which is approximately 30 nm for Fe_3_O_4_ nanoparticles [56]. However, in the case of pseudo-one-dimensional nanostructures such as nanotubes, we almost always face a single-domain phase due to the large aspect ratio of this nanostructure. In this article, we consider a length of 1000 nm because Xiang et al. [57] have shown that the coercivity of a 20 nm diameter Fe nanowire does not vary for lengths greater than 200 nm. In fact, to ensure that this behavior is also valid for Fe_3_O_4_, we simulated 30 nm diameter nanowires with three different hole sizes, *β* = 0.1, 0.5, and 0.9, with lengths ranging between 1000 and 5000 nm (see Figure A1 of Appendix A). We considered two possible external radii, *R_ext_* = 30 and 80 nm, and a variable internal radius, *r_int_*, which allowed us to define the ratio *β* = *r_int_*/*R_ext_* such that *β* = 0 represents a solid cylinder and *β* → 1 corresponds to a very narrow tube (Figure 1). According to Escrig et al. [16], 80 nm diameter nanotubes are wide enough to reverse their magnetization through the nucleation and propagation of vortex domain walls, whereas thin 30 nm nanotubes can reverse their magnetization through transverse domain walls for small holes and vortex domain walls for large holes. Therefore, we have chosen well-differentiated diameters to see how this change in the reversal modes affects the static and dynamic magnetic properties of the nanotubes.

The magnetic parameters used for magnetite (Fe_3_O_4_) were the saturation magnetization M_s_ = 4.8 × 10^5^ A/m, the stiffness constant A = 2.64 × 10^−11^ J/m, and the cubic magnetocrystalline anisotropy K_c_ = −1.25 × 10^4^ J/m^3^ [58]. In the simulations, we used a cell size of 2 × 2 × 5 nm^3^, which is small enough to accurately capture the geometry of the nanostructures. To simulate the magnetic configurations of minimum energy and the hysteresis loops (static magnetic properties), we used a large value of α = 0.5, which is commonly used to reduce simulation time without significantly affecting the results of quasi-static simulations. The external magnetic field was applied parallel to the *z*-axis (θ = 0°) with a magnitude of H = 1000 mT to saturate the samples, and field steps of 2 mT were used to investigate the hysteresis curves.

On the other hand, to simulate the dynamic response of magnetization (FMR spectra), we used α = 0.015 [59]. In addition, we applied a sinc wave excitation field [51]

$$h_{sinc}=h_{0}\frac{sin\left(2\pi f_{c}\tau \right)}{2\pi f_{c}\tau }$$

along the *y*-direction to perturb the magnetization of the system. The sinc function is a mathematical function used to describe the spectral response of a low-pass filter or the pulse response of a sampling system. In this equation, $h_{0}$ = 5 mT is the amplitude of the sinc function at its peak, the cut-off frequency is $f_{c}$ = 45 GHz, and *τ* = t − t_0_ was the simulation time (t), with an offset t_0_. The data in the time domain were recorded for 20 ns with a step time of 10 ps, allowing for a better spectral resolution of 0.05 GHz. For FMR analysis, we derived the imaginary part of the dynamical susceptibility via a fast-Fourier transform (FFT) procedure. Specifically, the frequency-dependent magnetic susceptibility of a material, χ(ω), is defined as the ratio of the Fourier component, m_y_(ω), of the *y*-component of the spatially averaged magnetization m(t) and the Fourier component, h(ω), of the applied exciting field [60],

χ(ω) = m_y_(ω)/h(ω).

We further obtained the spatial FMR mode profiles from the post-processing of the position-dependent magnetization data. The magnetic susceptibility is a fundamental property of magnetic materials that determines how they respond to magnetic fields. It is used to characterize the magnetic properties of materials in a variety of applications, including magnetic storage, magnetic resonance imaging, and magnetic sensors. The equation is often used in the analysis of magnetic materials and in the design of magnetic devices.

## 3. Results and Discussion

In this section, we present and analyze the results of our micromagnetic simulations for the static and dynamic properties of Fe_3_O_4_ nanotubes. For the static behavior, we focus on the coercivity, remanence, and magnetization reversal modes. For the dynamic behavior, we analyze the dynamic susceptibility and the resonant frequencies of the peaks for the different geometric parameters considered. 

### 3.1. Static Magnetic Properties

**Figure 2:**
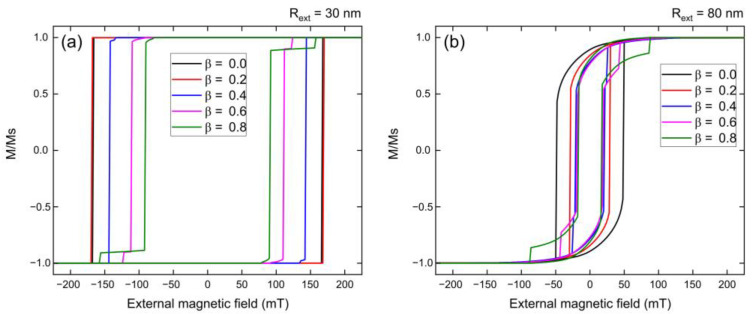
Hysteresis curves for 1000 nm long Fe_3_O_4_ nanotubes with external radius: (**a**) *R_ext_* = 30 nm and (**b**) *R_ext_* = 80 nm, for different *β* values, when the external magnetic field is applied along the *z*-axis.

**Figure A2:**
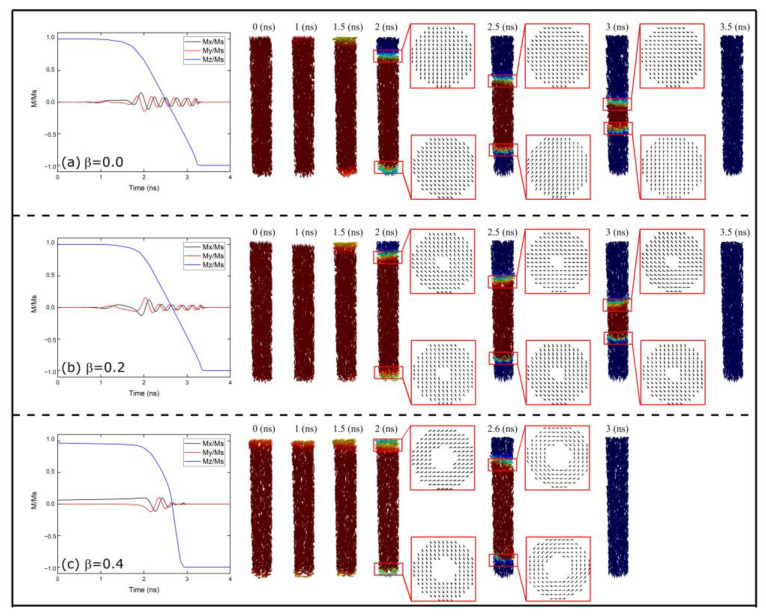
Magnetization reversal of a 1000 nm long Fe_3_O_4_ nanotube with an external radius of 30 nm, for *β* = 0.0 (**a**), 0.2 (**b**), and 0.4 (**c**). The graphs on the left depict the components of magnetization as a function of time, while the snapshots of magnetization at specific times are presented on the right of each graph.

In the first stage, we obtained the hysteresis curves of Fe_3_O_4_ nanotubes with a length of 1000 nm for two different external radii, *R_ext_* = 30 nm (Figure 2a) and *R_ext_* = 80 nm (Figure 2b), considering different tube walls by varying the *β* = *r_int_*/*R_ext_* value. The hysteresis curves shown in Figure 2a,b differ due to the use of different diameters (see Figure 3 of Ref. [57]). Figure 2a shows that nanotubes with an external radius of 30 nm present square hysteresis curves, with significant coercivities (Hc) and normalized remanences (Mr/Ms) very close to 1.0. The appearance of a small hole (*β* = 0.2) produces a slight increase in coercivity when compared to the coercivity produced by a nanowire (*β* = 0.0). However, for larger holes (*β* ≥ 0.4), the coercivity begins to decrease drastically. To explain this non-monotonic behavior, in Appendix A we plotted the magnetization reversal process of these nanotubes as a function of time (see Figure A2). From Figure A2a, we can see that a nanowire (*β* = 0.0) reverses its magnetization via nucleation and propagation of two transverse domain walls in the caps that propagate towards the center of the nanowire. Figure A2b shows that in the case of a nanotube with a small hole (*β* = 0.2), the system keeps reversing its magnetization via transverse domain walls. However, due to the new inner surface, the magnetic moments are not perfectly aligned, resulting in a slight increase in coercivity. Figure A2c shows that a nanotube with a larger hole (*β* = 0.4) nucleates transverse domain walls at the caps, which, as they propagate towards the center of the nanotube, become vortex walls, speeding up the magnetization reversal process and allowing a decrease in the coercivity of the system. It is also interesting to note that a step appears in the hysteresis curve for larger holes, whose size increases as the size of the hole increases. A similar behavior where nanotubes reverse their magnetization through different reversal mechanisms as a function of the tube wall width was previously observed [16,17].

**Figure A3:**
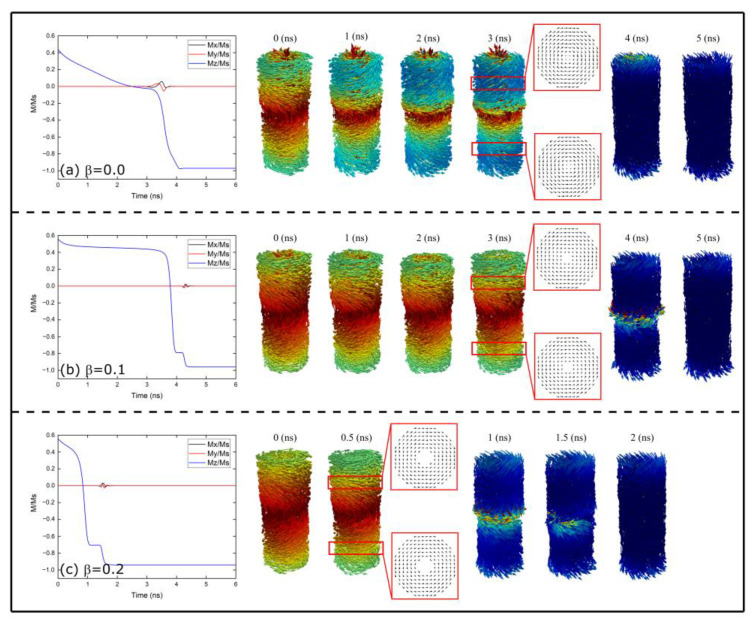
Magnetization reversal of a 1000 nm long Fe_3_O_4_ nanotube with an external radius of 80 nm, for *β* = 0.0 (**a**), 0.1 (**b**), and 0.2 (**c**). The graphs on the left depict the components of magnetization as a function of time, while the snapshots of magnetization at specific times are presented on the right of each graph.

In contrast, Figure 2b shows that Fe_3_O_4_ nanotubes with an external radius of 80 nm exhibit more rounded hysteresis curves, with lower coercivities (Hc) and normalized remanences (Mr/Ms) compared to those observed for nanotubes with an external radius of 30 nm. Additionally, it is observed that these larger nanotubes also display a step in their hysteresis curve, for practically all values of *β*. To explain these behaviors, we have graphed the magnetization reversal process of these nanotubes as a function of time in Appendix A. From Figure A3a–c, we observe that both nanowire (*β* = 0.0) and nanotubes (*β* = 0.1 and 0.2) reverse their magnetization by nucleating and propagating two vortex domain walls at the caps that propagate towards the center of the nanostructure. This implies that for this outer radius, there is a unique magnetization reversal mechanism, which is why the coercivity decreases monotonically as we increase the value of *β*. Furthermore, Figure A3b,c indicate that the steps that appear in the hysteresis curves are due to the collision between the two vortices with different chiralities in the center of the nanotube, which requires a larger field for complete annihilation.

**Figure 3:**
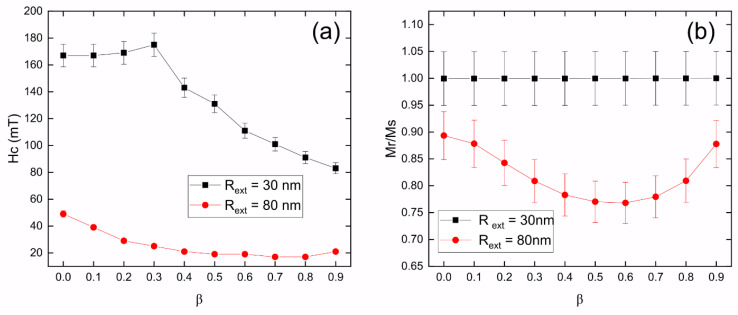
(**a**) Coercivity and (**b**) normalized remanence for 1000 nm long Fe_3_O_4_ nanotubes with external radius 30 nm (black squares) and 80 nm (red circles), as a function of *β*, when the external magnetic field is applied along the *z*-axis. We considered an error of 5%.

Figure 3 displays the coercivity and normalized remanence for 1000 nm long Fe_3_O_4_ nanotubes with an external radius of 30 nm and 80 nm, as a function of *β*, when the external magnetic field is applied along the *z*-axis. From Figure 3a, it is evident that the coercivity of nanotubes with an external radius of 30 nm is consistently higher that the coercivity of nanotubes with an external radius of 80 nm, irrespectively of the value of *β*. Additionally, we observe that the coercivity of nanotubes with an external radius of 30 nm follows a non-monotonic behavior, reaching a maximum value close to 180 mT for *β* = 0.3, and beyond which it starts decreasing until reaching approximately 80 mT for *β* = 0.9. This non-monotonic behavior is due to the fact that nanotubes with *β* < 0.4 reverse their magnetization through transverse domain walls, while nanotubes with *β* ≥ 0.4 reverse their magnetization through vortex domain walls. From Figure 3b, we see that these nanotubes with an external radius of 30 nm exhibit a normalized remanence equal to 1.0 for all *β* values, which explains the square behavior of the hysteresis curves shown in Figure 2a.

Figure 3a indicates that the coercivity of 80 nm outer radius nanotubes decreases as a function of *β*, from approximately 50 mT for *β* = 0.0 to almost 20 mT for *β =* 0.8. This can be explained by the fact that these nanotubes reverse their magnetization through the nucleation and propagation of vortex domain walls. On the other hand, Figure 3b shows that the normalized remanence exhibits a non-monotonic behavior as a function of *β*, with values close to 0.9 for *β* = 0.0 and 0.9, and a value that reduces to a valley close to 0.75 for *β* = 0.6. This explains the more rounded behavior exhibited by the hysteresis curves shown in Figure 2b.

### 3.2. Dynamic Magnetic Properties

**Figure 4:**
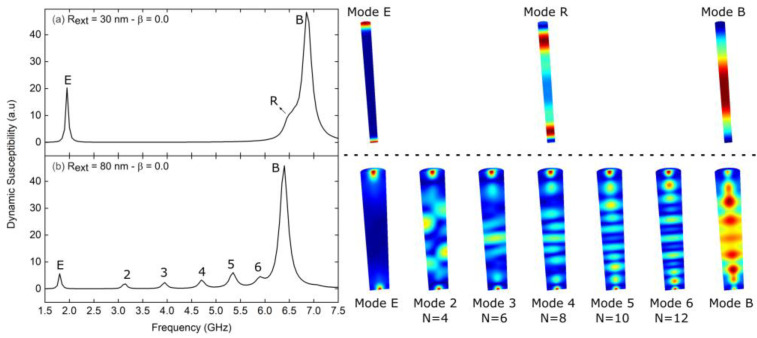
Dynamic susceptibility spectra for 1000 nm long Fe_3_O_4_ nanotubes with external radius (**a**) *R_ext_* = 30 nm and (**b**) *R_ext_* = 80 nm, for *β* = 0.0, while the system is excited with a sinc wave field in the *y*-direction. On the right are the spatial profiles of the resonant modes shown to the left. The color scale corresponds to the amplitude of the magnetic moment fluctuations, with red depicting the largest amplitude and blue denoting zero amplitude.

**Figure A4:**
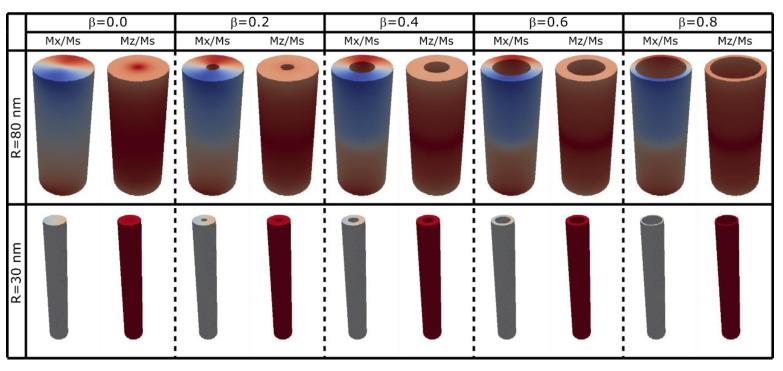
Components of the magnetization (Mx/Ms and Mz/Ms) for each of the geometries that were excited to investigate the FMR spectrum of the 1000 nm long Fe_3_O_4_ nanotubes.

**Figure A5:**
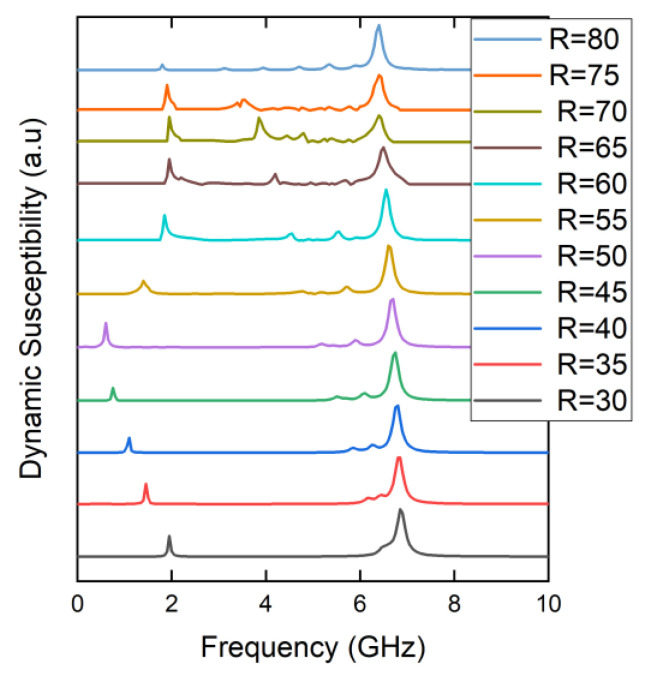
Dynamic susceptibility spectra for 1000 nm long Fe_3_O_4_ nanotubes with different radii ranging between 30 and 80 nm, for different *β* values, while the system is excited with a sinc wave field in the *y*-direction.

To investigate the magnetization dynamics of 1000 nm long Fe_3_O_4_ nanotubes, we carried out ferromagnetic resonance (FMR) studies on the remanent state of each geometry (see Figure A4 in Appendix A). Subsequently, we investigated the dynamic susceptibility of nanotubes with an external radius of 30 and 80 nm by applying a sinc wave excitation in the *y*-direction. Figure 4a shows the resonant modes for the nanowire (*β* = 0.0) with an external radius of 30 nm. We can observe two well-defined modes, one of low frequency mode edge (mode E), associated with the caps of the nanowire, and another of high frequency mode bulk (mode B), of greater height, as it excites a larger number of magnetic moments associated with the central area of the nanowire. The latter presents a small bump that we have defined as the mode ring (mode R), which is associated with a disturbance close to the edges of the wire. On the other hand, Figure 4b shows the resonant modes for the nanowire (*β* = 0.0) with an external radius of 80 nm. In this case, we can see that the number of modes increases, where seven modes are clearly identified, and among which we can again highlight the low frequency mode, mode E, and the high frequency mode, mode B. In this last mode, the bump we called mode R does not appear, but we can observe a similar behavior in mode 6. The increase in the number of modes that occurs when we go from a thin nanotube to a thick one is expected. Lupo et al. [61] investigated the radial and azimuthal resonance modes of spin waves in Ni_80_Fe_20_ (permalloy) nanodisks in the absence of an external magnetic field, finding that the number of absorption modes increased as the disk diameter increased. A similar conclusion was reached by Dobrovolskiy et al. [62], who investigated Co-Fe nanodisks. In fact, we have shown in Figure A5 of Appendix A that the number of resonance modes increases as the diameter of the nanotubes increases. It is evident from the FFT power distributions that each mode can be characterized by the number of nodes (N) present along the longitudinal axis of the nanotube [63].

**Figure 5:**
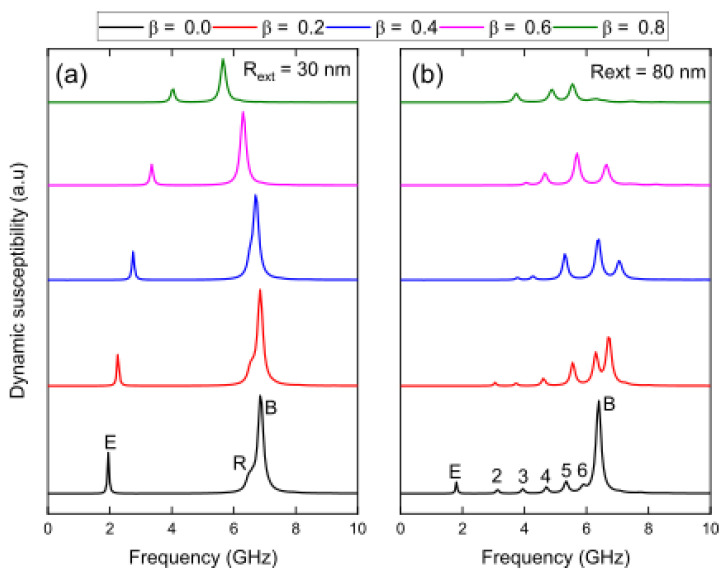
Evolution of dynamic susceptibility spectra for 1000 nm long Fe_3_O_4_ nanotubes with external radius (**a**) *R_ext_* = 30 nm and (**b**) *R_ext_* = 80 nm, as a function of *β* values. The system is excited with a sinc wave field in the *y*-direction.

In Figure 5, we can observe how these modes change with respect to *β*. In Figure 5a, it is shown that mode R, which corresponds to a bump of mode B, almost disappears for *β* ≥ 0.4. This implies that nanotubes with an external radius of 30 nm with large holes only exhibit two resonance modes, namely mode E and mode B. As the value of *β* increases, both modes move closer towards intermediate frequencies. In Figure 5b, it is noticeable that the seven modes that appear for *β* = 0.0 change remarkably, with only modes E, 2, 3, and 4 surviving for *β* = 0.8. In fact, the mode that stands out the most for *β* = 0.0, mode B, only survives for small *β* values.

**Figure 6:**
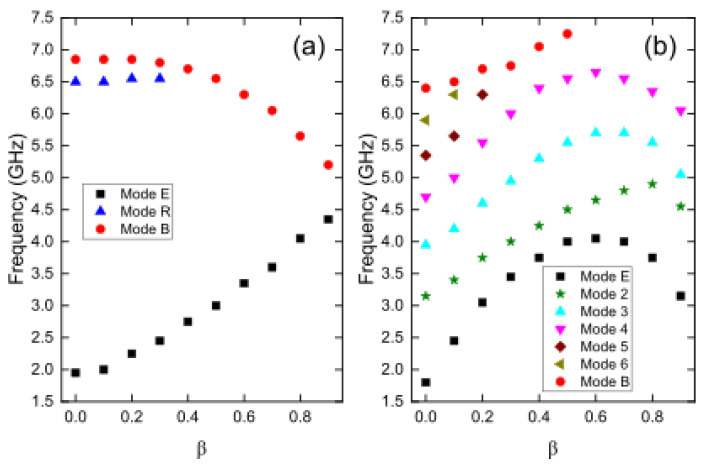
Evolution of resonance modes for 1000 nm long Fe_3_O_4_ nanotubes with external radius (**a**) *R_ext_* = 30 nm and (**b**) *R_ext_* = 80 nm, as a function of the parameter *β*.

Figure 6 shows the evolution of resonance modes for Fe_3_O_4_ nanotubes of length 1000 nm and external radii of 30 and 80 nm, as a function of the parameter *β*. It can be observed from this figure that mode E increases monotonically from approximately 2.0 GHz for *β* = 0.0 to 4.5 GHz for *β* = 0.9 when the external radius is 30 nm. However, when the nanotube has an external radius of 80 nm, the same resonance mode follows a non-monotonic behavior, reaching a maximum value of approximately 4.0 GHz for *β* = 0.6. Mode B monotonically decreases from approximately 7.0 GHz for *β* = 0.0 to 5.0 GHz for *β* = 0.9 when the external radius is 30 nm, while this mode experiences a sustained increase when the external radius is 80 nm, disappearing for *β* ≥ 0.6. Figure 6a also shows that mode R remains stable around 6.5 GHz for *β* ≤ 0.3, while modes 2, 3, and 4 observed in Figure 6b follow a similar non-monotonic behavior as mode E, and modes 5 and 6, grow monotonically with *β* but do not exist for the entire investigated range, similar to what occurs with mode B.

## 4. Conclusions

In summary, this study used micromagnetic simulations to investigate the static and dynamic magnetic properties of Fe_3_O_4_ nanotubes with varying external radii and tube wall thicknesses. Our findings revealed that the coercivity and remanence of the nanotubes are non-zero when a magnetic field is applied parallel to the tube axis. The coercivity of the 30 nm nanotubes exhibited a non-monotonic behavior, while the coercivity of the 80 nm nanotubes decreased monotonically with increasing β. We also found that the geometry of the nanotubes can be used to control the number of resonance modes and their frequencies, making them a potential candidate for use in microwave devices.

Overall, this study contributes to the understanding of the magnetic properties of Fe_3_O_4_ nanotubes and demonstrates the potential for their use in practical applications.

## Data Availability

Data will be made available upon request.
